# Delayed skin reactions after the second dose of mRNA vaccines against SARS-CoV-2

**DOI:** 10.17179/excli2022-5216

**Published:** 2022-07-27

**Authors:** Paulo Ricardo Martins-Filho, Martha Débora Lira Tenório

**Affiliations:** 1 Investigative Pathology Laboratory, Federal University of Sergipe, Aracaju, SE, Brazil; 2 Dermatology Residence Program, Federal University of Sergipe, Aracaju, SE, Brazil

## ⁯⁯

Studies have evaluated the incidence rate of delayed local reactions (DLR) among individuals who received the first dose of the mRNA-1273 vaccine against SARS-CoV-2. In a more recent study published in Japan (Higashino et al., 2022[6]), it was shown an overall incidence of DLR of 12.7 %, with higher rates among females and individuals aged 30 to 69 years. The incidence reported in this study was higher than that found in observational studies conducted in the United States (1.1 %) (Jacobson et al., 2022[7]) and Spain (2.1 %) (Fernandez‐Nieto et al., 2021[3]), which can be explained by differences in surveillance systems and diagnostic criteria. However, despite the advancement of COVID-19 vaccination worldwide, little has been discussed about the occurrence of DLR after the second dose of mRNA vaccines and recurrence rates. The occurrence of DLR can influence the patient's decision to complete their vaccination schedule.

Here, we evaluate the available evidence (through June 17, 2022) on the occurrence of DLR following the second dose of mRNA vaccines (BNT162b2 or mRNA-1273) against SARS-CoV-2. Studies with samples smaller than 25 patients were excluded. Delayed skin reactions and second-dose recurrence data were extracted. The overall proportion of DLR after the second dose and recurrence rates were calculated using the variance-stabilizing Freeman-Tukey double-arcsine transformation with an inverse-variance random-effects model. Analyses were conducted in RStudio (version 0.98.1083) following the Preferred Reporting Items for Systematic Reviews and Meta-Analyses (PRISMA) guideline (Moher et al., 2010[12]). 

We found nine studies that met the eligibility criteria by conducting a systematic search on PubMed, Embase, and Scopus using the keywords "*delayed local reactions*," "*COVID-19*," and "*mRNA SARS-CoV-2 vaccines*" and related terms. A total of 1,334 individuals reported the occurrence of DLR, of which 354 were registered after the second dose of mRNA vaccines. The proportion of DLR ranged from 13.0 % (95 % CI 6.4 - 22.6) to 52.4 % (95 % CI 42.4 - 62.4) and the between-study heterogeneity was considered high (I^2^ = 91.6 %). The overall proportion of DLR after the second dose of mRNA vaccines was 29.4 % (95 % CI 19.8 - 40.0). Five studies reported 82 cases of second-dose recurrence and the overall rate was 24.8 % (95 % CI 10.4 - 42.8) with high between-study heterogeneity (I^2^ = 93.7 %) (Table 1(Tab. 1); References in Table 1: Baden et al., 2021[1]; Català et al., 2022[2]; Fernandez-Nieto et al., 2021[3]; Freeman et al., 2022[4]; Hibino et al., 2021[5]; Juárez Guerrero et al., 2021[9]; Kitagawa et al., 2022[10]; McMahon et al., 2021[11]; Papadimitriou et al., 2022[13]). 

Available evidence suggests that approximately one third of cases of DLR may occur after the second dose of mRNA vaccines, but this rate may be underestimated due to potential reporting bias. Moreover, recurrent reactions are not uncommon and can be found in about 25 % of patients after the first dose. Since most cases are mild and self-limiting, and likely associated with lymphocytes and eosinophils infiltration at the site of vaccine application (Johnston et al., 2021[8]), there is no absolute contraindication to the use of mRNA vaccines in patients with a history of DLR. However, patients need to be educated about the benefits and side effects of vaccines to prevent vaccine hesitancy. 

## Declaration

### Authors' contributions

All authors contributed equally to this work.

### Conflict of interest statement

The authors have no conflicts of interest to declare.

### Role of funding source

There is no funding source.

## Figures and Tables

**Table 1:**
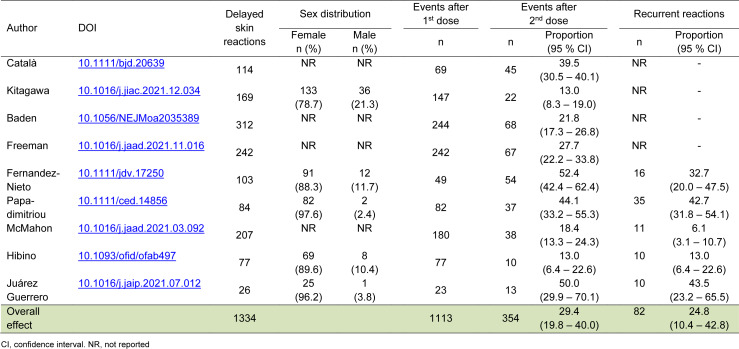
Studies reporting delayed local reactions after the second dose of mRNA vaccines against SARS-CoV-2
